# Reconstructing Dynamic Gene Regulatory Networks Using f-Divergence from Time-Series scRNA-Seq Data

**DOI:** 10.3390/cimb47060408

**Published:** 2025-05-30

**Authors:** Yunge Wang, Lingling Zhang, Tong Si, Sarah Roberts, Yuqi Wang, Haijun Gong

**Affiliations:** 1 Department of Mathematics and Statistics, Saint Louis University, St. Louis, MO 63103, USA; yunge.wang@slu.edu (Y.W.); sarah.roberts@slu.edu (S.R.); 2 Department of Mathematics and Statistics, University at Albany SUNY, Albany, NY 12222, USA; lzhang28@albany.edu; 3 Mathematics Department, Culver-Stockton College, Canton, MO 63435, USA; tsi@culver.edu; 4 Department of Biology, Saint Louis University, St. Louis, MO 63103, USA; yuqi.wang@slu.edu

**Keywords:** gene regulatory network, time-varying network, single-cell RNA sequencing, time-series data, f-divergence, Granger causality, regularization

## Abstract

Inferring time-varying gene regulatory networks from time-series single-cell RNA sequencing (scRNA-seq) data remains a challenging task. The existing methods have notable limitations as most are either designed for reconstructing time-varying networks from bulk microarray data or constrained to inferring stationary networks from scRNA-seq data, failing to capture the dynamic regulatory changes at the single-cell level. Furthermore, scRNA-seq data present unique challenges, including sparsity, dropout events, and the need to account for heterogeneity across individual cells. These challenges complicate the accurate capture of gene regulatory network dynamics over time. In this work, we propose a novel f-divergence-based dynamic gene regulatory network inference method (f-DyGRN), which applies f-divergence to quantify the temporal variations in gene expression across individual single cells. Our approach integrates a first-order Granger causality model with various regularization techniques and partial correlation analysis to reconstruct gene regulatory networks from scRNA-seq data. To infer dynamic regulatory networks at different stages, we employ a moving window strategy, which allows for the capture of dynamic changes in gene interactions over time. We applied this method to analyze both simulated and real scRNA-seq data from THP-1 human myeloid monocytic leukemia cells, comparing its performance with the existing approaches. Our results demonstrate that f-DyGRN, when equipped with a suitable f-divergence measure, outperforms most of the existing methods in reconstructing dynamic regulatory networks from time-series scRNA-seq data.

## 1. Introduction

A comprehensive understanding of gene regulatory networks (GRNs) is essential for uncovering the mechanisms governing cellular processes, biological functions, cell differentiation, and disease progression. Accurate inference of these networks could significantly contribute to the advancement of precision medicine and the development of targeted therapeutic strategies for a wide range of diseases. However, reconstruction of GRNs from genome-wide sequencing data is a challenging problem in computational and systems biology research. Various computational approaches [1,2,3,4,5,6] have been developed for GRN inference from bulk RNA or microarray data, including deterministic methods, such as Boolean networks [7,8], differential equations [9,10,11], and matrix factorization [12,13], as well as probabilistic graphical models, including Bayesian networks (BNs) [14,15,16], dynamic Bayesian networks (DBNs) [17,18,19], graphical LASSO [20,21], Granger causality methods [22], the tree-based Jump3 method [23], and TSNI [10] for time-series data. Recently, a transformer-based method called TRENDY [24] was proposed for regulatory network inference. Methods based on bulk RNA or microarray data have substantially advanced our understanding of gene regulatory mechanisms at the tissue level.

Recent advancements in single-cell profiling technologies, such as single-cell RNA sequencing (scRNA-Seq) [25], quantitative RT-PCR (qRT-PCR) [26], and single-cell ChIP-seq (scChIP-seq) [27], have revolutionized genomics studies by enabling gene expression profiling at a single-cell resolution. The analysis of scRNA-Seq data provides insights into cellular differentiation and heterogeneity across diverse populations, enabling the identification of cell-type-specific regulatory dynamics and key events that govern cell fate decisions. Ultimately, this paves the way for transformative advancements in disease modeling and the development of personalized medicine. Time-series scRNA-seq data provide richer temporal information compared to static data, making them more informative for regulatory network inference [3,28]. However, inferring gene regulatory networks from time-series scRNA-Seq data remains highly challenging due to their high dimensionality, inherent cellular heterogeneity [29], and substantial missing values caused by dropout events [30], which are attributed to technical limitations. Additionally, the limited number of time points further complicates the analysis of time-series scRNA-Seq data and the reconstruction of regulatory networks, potentially undermining the accuracy and robustness of the inferred networks.

Various inference algorithms have been developed to reconstruct gene regulatory networks from time-series single-cell RNA sequencing data. These include Boolean models [31,32], differential-equation-based approaches [33,34], and both supervised and unsupervised learning methods [5,35,36,37]. Boolean models and differential-equation-based approaches have been extensively reviewed by Nguyen et al. [38]. In this work, we will briefly review some popular methods for GRN inference from scRNA-seq data. The single-cell network synthesis (SCNS) toolkit [31] and BoolTraineR (BTR) [32] are two Boolean-network-based algorithms for GRN inference. SCNS constructs an asynchronous Boolean model by analyzing trajectories within a state transition graph, while BTR employs a scoring function grounded in the Boolean framework to infer network structures. These Boolean-network-based approaches require binarization of the data, which can lead to significant information loss. Moreover, their computational complexity increases significantly as the numbers of genes and cells grow. Differential-equation-based methods represent another class of continuous data approaches. For example, SCODE [33] and SCOUP [34] utilize ordinary and stochastic differential equations, respectively, to compute gene correlations and infer networks. GREMA [39] determines network regulations using a non-linear ODE model, which employs an evolutionary modeling algorithm to solve either an S-system or a Hill-function-based ODE model and finally identify the regulatory interactions.

Machine-learning-based network inference methods encompass correlation networks, regression-based approaches, as well as supervised and unsupervised learning techniques. Correlation-based methods assume that genes with correlated expression patterns are likely to be functionally related or regulated by the same transcription factors. These methods, such as the partial information decomposition and context (PIDC) method [35], analyze pairwise relationships between genes to construct undirected graphs. Although they are computationally efficient and scalable to large datasets, they do not capture causal relationships or directionality in gene regulation. Regression-based approaches infer the relationships between regulators and target genes by solving regression equations. To address the high dimensionality of gene expression data and sparsity of networks, various regularization techniques, such as LASSO (least absolute shrinkage and selection operator) and ridge regression, are commonly employed. For instance, the SINCERITIES method [40] uses a linear regression model to infer regulatory interactions. Additionally, methods like GENIE3 [5] and GRNBoost2 [36] utilize tree-based regression models and gradient boosting machines, respectively, to reconstruct gene regulatory networks. While regression-based methods offer interpretability and scalability, they often assume linear relationships, which may limit their ability to capture complex gene interactions. Several neural-network-based deep learning methods have been developed to address the inherent complexity of scRNA-seq data, including DeepMAPS [41], DeepSEM [37], DeepDRIM [42], 3DCEMA [43], and scTIGER [44], along with deep-generative-model-based approaches such as the hypergraph variational autoencoder (HyperG-VAE) [45] and Granger using causal recurrent autoencoders [46]. Recently, a quantum circuit model [47] was proposed to infer gene regulatory networks. However, deep learning methods require very large training datasets and are less interpretable due to the high number of parameters in neural networks compared to other machine learning models. Additionally, they struggle to effectively handle short-time-series datasets.

Most of these network inference approaches assume that the network structure is stationary. However, biological systems, including cell development, differentiation, and disease progression, are inherently dynamic, continuously evolving in response to various stimuli, stages, or conditions. Therefore, it is crucial to track the evolution of network structure over time. Inferring time-varying regulatory networks can enhance our understanding of the underlying mechanisms driving these processes and aid in identifying potential therapeutic interventions for disease treatment. To address this, various time-varying network inference methods have been proposed, including the dynamic vector-autoregressive model [48,49], heterogeneous and weighted dynamic Bayesian network model  [50,51,52,53], undirected L1-regularized logistic regression method [54], hidden Markov models [55], ARACNE algorithm based on mutual information [56], dynamic autoregressive Gaussian graphical model [57], time-lagged regression and ordered LASSO models [58,59], and time-varying graphical LASSO methods [60,61]. These methods can infer various types of time-varying genetic networks, including undirected graphs, correlation graphs, causal networks, and regulatory networks. However, these approaches are designed for bulk microarray data and fail to capture the temporal dynamics essential for understanding regulatory processes at the single-cell level. Recently, two ATAC-seq-based methods, Dictys and CellOracle [62,63], were developed to infer dynamic gene regulatory networks (GRNs). However, these methods are specifically designed for ATAC-seq data and are not directly applicable to time-series scRNA-seq data.

To address the limitations of the existing methods, our recent work introduced a KL-divergence-based approach for dynamic network inference [64]. Building on this, we now propose a more general framework, f-DyGRN (f-divergence-based dynamic gene regulatory network), designed to infer time-varying gene regulatory networks from time-series scRNA-seq data. In the Section 2, we outline the key components of our framework. First, we describe how f-divergence is utilized to estimate temporal variations in gene expression across individual single cells. Next, we introduce a first-order Granger causality model for directed network inference. To ensure sparsity in the inferred network, we incorporate various regularization techniques, including LASSO, MCP (minimax concave penalty), and SCAD (smoothly clipped absolute deviation penalty), to capture slowly changing sparse networks. Additionally, we integrate partial correlation analysis to identify regulatory relationships. Finally, we present an algorithm that integrates these components to reconstruct dynamic gene regulatory networks from time-series scRNA-seq data. We then apply our method to both simulated scRNA-seq data and real scRNA-seq data from THP-1 human myeloid monocytic leukemia cells, comparing the performance using different f-divergence. Finally, we discuss the advantages of our method and highlight the remaining challenges.

## 2. Materials and Methods

Given a time-series single-cell RNA sequencing (scRNA-seq) dataset consisting of *m* genes and *n* time points, the number of cells at each time point $t_{l}$ (l=1,2,…,n) is denoted as $s_{t_{l}}$. This time-series scRNA-seq dataset can be represented as a collection of gene expression matrices at each time point $t_{l}$: $X\in R^{s_{t_{l}}\times m}$, so each matrix *X* contains the expression levels of *m* genes across $s_{t_{l}}$ individual cells at time point $t_{l}$. These single-cell time-series gene expression matrices serve as the foundation for reconstructing time-varying gene regulatory networks.

### 2.1. f-Divergence-Based Temporal Variation Estimation

Previous studies, such as SINCERITIES [40], assume that changes in the expression level of a transcription factor directly influence the expression levels of its target genes. While these changes are relatively straightforward to compute in microarray data, scRNA-seq data necessitate a more sophisticated metric to quantify temporal variations at the single-cell level.

In this work, we assume that, at each time point *t*, the expression levels of a specific gene across all single cells follow a probability distribution. Therefore, temporal variations in gene expression across all cells can be quantified by measuring the divergence or dissimilarity between these distributions at consecutive time points. SINCERITIES [40] employed the Kolmogorov–Smirnov (KS) distance as a measure of distributional differences in gene expression over time. The KS distance quantifies the maximum absolute difference between two cumulative distribution functions (CDFs) and is defined as

$$\begin{array}{c} D_{KS}^{j}\left(l\right)=\max\left|F_{t_{l+1}}\left(X^{j}\right)-F_{t_{l}}\left(X^{j}\right)\right|, \end{array}$$

where $X^{j}$ represents the expression levels of gene *j*, and $F_{t_{l}}\left(X^{j}\right)$ denotes the CDF of gene *j*’s expression at time $t_{l}$. Thus, $D_{KS}^{j}\left(l\right)$ is the maximum distributional shift in gene *j*’s expression between consecutive time points $t_{l}$ and $t_{l+1}$.

However, since the KS distance only considers the maximum discrepancy in the cumulative distribution functions and does not account for differences across the entire distribution, it may fail to capture gradual shifts in gene expression over time. Additionally, scRNA-seq data are inherently sparse due to dropout events, so the KS distance is particularly sensitive to missing values since it relies on the empirical CDF. Although the KS distance is effective in detecting major shifts, it is less suitable for capturing gradual variations in the overall distribution of gene expression. Our recent work [64] replaced the Kolmogorov–Smirnov (KS) distance with Kullback–Leibler (KL) divergence, demonstrating promising performance.

An alternative solution is to use f-divergence, which can overcome some of the limitations of the KS distance in estimating the gene expression variation across all single cells. Further, f-divergence measures the difference between entire probability distributions rather than just the maximum difference, providing a more comprehensive assessment of distributional changes in gene expression dynamics.

Moreover, *f*-divergence measures the dissimilarity between two probability distributions *P* and *Q*, which is defined as

(1) $$\begin{array}{c} D_{f}\left(P||Q\right)=E_{x∼q}\left[f\left(\frac{p(x)}{q(x)}\right)\right]=\int_{\Omega }q\left(x\right)f\left(\frac{p(x)}{q(x)}\right)dx, \end{array}$$

where p(x) and q(x) are two probability density functions of *P* and *Q* defined over the domain Ω, and f(u) is a proper, lower semi-continuous, and convex function with the property f(1)=0.

With various choices regarding the function f(u), we can derive several well-known divergence measures. For example, the forward and backward (reverse) Kullback–Leibler (KL) divergences can be used to describe the temporal variation in scRNA-seq data and are expressed as

$$\begin{array}{cc} D_{\mathrm{KL}}^{j}\left(t_{l}\right) & =D_{\mathrm{KL}}\left(p^{j}\left(x,t_{l+1}\right)\left|\right|p^{j}\left(x,t_{l}\right)\right)=\int_{\Omega }p^{j}\left(x,t_{l+1}\right)\log\left(\frac{p^{j}\left(x,t_{l+1}\right)}{p^{j}\left(x,t_{l}\right)}\right)dx,\\ D_{\mathrm{rKL}}^{j}\left(t_{l}\right) & =D_{\mathrm{KL}}\left(p^{j}\left(x,t_{l}\right)\left|\right|p^{j}\left(x,t_{l+1}\right)\right)=\int_{\Omega }p^{j}\left(x,t_{l}\right)\log\left(\frac{p^{j}\left(x,t_{l}\right)}{p^{j}\left(x,t_{l+1}\right)}\right)dx, \end{array}$$

where $p^{j}\left(x,t_{l}\right)$ and $p^{j}\left(x,t_{l+1}\right)$ represent the probability density function (PDF) that gene *j*’s expression levels across all single cells follow at times $t_{l}$ and $t_{l+1}$, respectively. Both the forward and reverse KL divergences are asymmetric. To obtain a symmetric measure, we define the symmetric KL divergence as the average of the two:

$$D_{\text{S-KL}}^{j}\left(t_{l}\right)=\frac{1}{2}\left(D_{\mathrm{KL}}^{j}\left(t_{l}\right)+D_{\mathrm{rKL}}^{j}\left(t_{l}\right)\right).$$

Another type of symmetric KL-based divergence is the Jensen–Shannon (JS) divergence, which measures the KL divergence between each distribution and their averaged mixture distribution *M*, defined as

$$M=\frac{P+Q}{2}=\frac{p^{j}\left(x,t_{l+1}\right)+p^{j}\left(x,t_{l}\right)}{2}.$$

Mathematically, the JS divergence is given by

$$D_{\mathrm{JS}}^{j}\left(t_{l}\right)=\frac{1}{2}D_{\mathrm{KL}}(p^{j}\left(x,t_{l+1}\right)\left||M\right)+\frac{1}{2}D_{\mathrm{KL}}(p^{j}\left(x,t_{l}\right)||M),$$

where $D_{\mathrm{KL}}(p^{j}\left(x,t_{l+1}\right)\left||M\right)$ and $D_{\mathrm{KL}}(p^{j}\left(x,t_{l}\right)\left||M\right)$ denote the KL divergences between $p^{j}\left(x,t_{l+1}\right)$ and *M* and $p^{j}\left(x,t_{l}\right)$ and *M*, respectively. Unlike KL divergence, JS divergence is always symmetric, making it more robust in certain applications.

Table 1 summarizes several f-divergence functions that are used to estimate the temporal variation in scRNA-seq data in this work, including KL divergence and Pearson divergence, as well as the symmetric divergence functions based on the KL and Pearson divergence. Our recent studies [65,66] in missing value imputation have shown that f-divergence provides flexibility in learning the dissimilarities between distributions. Compared to SINCERITIES’ use of the Kolmogorov–Smirnov (KS) distance [40] and our recent work employing KL divergence [64], the f-divergence-based approach offers greater flexibility in capturing distributional changes. Unlike the KS distance, which considers only the maximum discrepancy at a single point, f-divergence accounts for differences across the entire distribution, making it more robust for detecting subtle and gradual shifts in gene expression over time.

In our subsequent calculations, we use $D_{f}^{j}\left(t_{l}\right)$ to represent the f-divergence, which describes the temporal variations in the gene *j*’s expression levels across all single cells between two consecutive time points, $t_{l}$ and $t_{l+1}$. Since the time points in the scRNA-seq data are sampled non-uniformly, we adopt the normalization strategy from SINCERITIES [40]; $D_{f}^{j}\left(t_{l}\right)$ is normalized with respect to the time window size as follows:

(2) $${\hat{D}}_{f}^{j}\left(t_{l}\right)=\frac{D_{f}^{j}\left(t_{l}\right)}{\Delta t_{l}},$$

where $\Delta t_{l}=t_{l+1}-t_{l}$ represents the time interval between two consecutive measurements. This normalization ensures that divergence values are comparable across different time intervals, mitigating biases introduced by non-uniform sampling.

### 2.2. Granger Causality for Directed Network Inference

Granger causality [67] is a statistical method to infer directed causal relationships in multivariate time-series data by identifying edges between variables based on their temporal dependencies. Given an *m*-dimensional vector, $X\left(t\right)={\left[X_{1}\left(t\right),X_{2}\left(t\right),...,X_{m}\left(t\right)\right]}^{T}$, representing the values of *m* variables at time *t*, a linear vector autoregressive (VAR(*p*)) model is used to model the Granger causality with *p* time lags, which is formulated as

$$X\left(t\right)=A_{1}X\left(t-1\right)+A_{2}X\left(t-2\right)+\ldots +A_{p}X\left(t-p\right)+\epsilon_{t},$$

where $A_{k}$ (for k=1,2,…,p) are m×m autoregressive coefficient matrices, and each matrix captures the dependencies between variables at different time lags; $\epsilon_{t}$ represents the noise in the model.

In many real-world time-series data analyses, the most recent observations tend to be more predictive of future states than older ones. This implies that, as the lag *k* increases, the influence of X(t−k) on X(t) gradually diminishes. In this work, we apply a first-order Granger causality model to infer causal relationships between genes. This choice helps to reduce model complexity and mitigate overfitting that may arise from incorporating higher-order lags. In this model, the prediction of X(t) depends on only the most recent observation X(t−1), and the causal relationship is learned using a first-order vector-autoregressive model VAR(1):

(3) $$X\left(t\right)=AX\left(t-1\right)+\epsilon_{t}.$$

The matrix *A* captures the dependencies between variables. If an entry $A_{ij}$ in the matrix *A* is significantly different from zero, it indicates that variable $X_{j}$ Granger-causes variable $X_{i}$, implying a directed edge $X_{j}\to X_{i}$ in the inferred network. Similar to previous work [40,64], we formulate the GRN inference problem as predicting the shift in the expression distribution of a target gene *j*, denoted as ${\hat{D}}_{f}^{j}\left(t_{l+1}\right)$ using f-divergence at time $t_{l+1}$. This prediction is based on the changes in the expression distributions of all genes at the current time $t_{l}$, denoted as ${\hat{D}}_{f}^{1:m}\left(t_{l}\right)$. This relationship is modeled using a first-order vector-autoregressive model (VAR(1)):

(4) $${\hat{D}}_{f}^{j}\left(t_{l+1}\right)=\sum_{p=1}^{m}\alpha_{p,j}{\hat{D}}_{f}^{p}\left(t_{l}\right)+\epsilon_{t},$$

where $\alpha_{p,j}$ quantifies the influence of gene *p* on gene *j*. A nonzero $\alpha_{p,j}$ indicates the presence of a directed edge from gene *p* to gene *j*, suggesting a potential regulatory interaction in the inferred GRN.

To capture the structural changes in gene regulatory networks across different stages, we employ a moving window approach to infer time-varying regulatory relationships. Specifically, we define a window of size *w* that slides over the time series of gene expression distributions ${\hat{D}}_{f}\left(t\right)$ for each gene. Within each window, we infer regulatory interactions by estimating the coefficients α in the regression model defined in Equation (4) based on the available data. By continuously sliding the window across the time series, we systematically track the changes in α, enabling us to model transient interactions and stage-specific regulations. This approach allows us to monitor the evolution of the network structure, which is often crucial in biological processes such as cell differentiation, disease progression, and responses to perturbations.

To obtain the optimal α, we minimize the squared error between the predicted and observed shifts in gene expression distributions. In matrix form, let ${\hat{D}}_{f}\left(t\right)\in R^{T\times m}$ represent the normalized temporal variation in all genes over *T* time points, and let $\alpha_{j}\in R^{m}$ be a vector of regression coefficients for gene *j*, describing the influence of other genes on gene *j*. The optimization problem for each gene *j* in each window can then be written as

(5) $$\min_{\alpha_{j}}{∥{\hat{D}}_{f}^{j}\left(t_{l+1}\right)-{\hat{D}}_{f}\left(t_{l}\right)\alpha_{j}∥}^{2}.$$

However, due to the high dimensionality of genes (m≫T) in the scRNA-seq data, the limited number of time points, and the sparsity of the regulatory network, directly solving the above optimization problem may result in overfitting and unstable estimates. To enforce sparsity in $\alpha_{j}$ and the inferred regulatory network, we incorporate regularization techniques, ensuring robust and biologically meaningful network structures. The regularized optimization problem is formulated as

(6) $$\min_{\alpha_{j}}\frac{1}{2}{∥{\hat{D}}_{f}^{j}\left(t_{l+1}\right)-{\hat{D}}_{f}\left(t_{l}\right)\alpha_{j}∥}^{2}+\lambda p\left(\alpha_{j}\right),$$

where $p(\alpha_{j})$ is a regularization function, λ controls the strength of regularization, and the sparse matrix α represents the inferred connectivity structure of the network at different stages.

Most network inference methods [40,58] apply bridge regression to solve the following optimization problem:

(7) $$\min_{\alpha_{j}}\frac{1}{2}∥{\hat{D}}_{f}^{j}\left(t_{l+1}\right)-{\hat{D}}_{f}\left(t_{l}\right)\alpha_{j}{∥}^{2}+\lambda {∥\alpha_{j}∥}^{q},$$

where q>0 controls the type of regularization applied.

LASSO [68], corresponding to q=1, enforces sparsity by shrinking many coefficients to exactly zero. Ridge regression, or the $L_{2}$-norm penalty, corresponds to q=2, shrinking coefficients toward zero, but does not enforce exact sparsity. These methods have been widely adopted in network inference, such as in SINCERITIES [40], to infer sparse regulatory networks. However, LASSO, due to its sharp shrinkage property, tends to produce significant structural changes between network stages, with sudden additions or removals of many edges in response to small variations in the data. In biological systems, however, regulatory networks typically evolve gradually [58] rather than undergoing drastic structural changes between adjacent time points.

### 2.3. Regularization Methods for Slowly Changing Sparse Network Inference

In time-varying network inference, the choice in regularization method is critical for accurately capturing both the sparsity and smooth evolution of the regulatory network. While LASSO is effective in enforcing sparsity by selecting a subset of relevant edges, its uniform shrinkage mechanism introduces bias, leading to potential distortions in the inferred network structure. Moreover, LASSO’s tendency to induce abrupt transitions between consecutive time points is not consistent with the expected gradual evolution of networks in a real biological system. To address the limitations of LASSO, in the current study, we further explore alternative regularization methods that can promote both sparsity and smooth transitions in time-varying network inference.

#### 2.3.1. Smoothly Clipped Absolute Deviation Penalty

The smoothly clipped absolute deviation (SCAD) penalty [69] is a regularization method specifically developed to overcome the limitations of LASSO. SCAD reduces the bias associated with large coefficient estimates while preserving sparsity in the model. The SCAD penalty function for each coefficient is defined as

(8) $$p_{\lambda }\left(\alpha \right)=\left\{\begin{array}{cc} \lambda |\alpha |, & \mathrm{if}\;|\alpha |\leq \lambda \\ \frac{2a\lambda |\alpha |-\alpha^{2}-\lambda^{2}}{2(a-1)}, & \mathrm{if}\;\lambda <|\alpha |\leq a\lambda \\ \frac{\left(a+1\right)\lambda^{2}}{2}, & \mathrm{if}\;|\alpha |>a\lambda , \end{array}\right.$$

where λ>0 is the regularization parameter controlling sparsity; a>2 (typically set to 3.7) determines the concavity of the penalty, meaning it controls how quickly the penalty diminishes for large values of |α|.

The first derivative of the SCAD penalty, which determines the shrinkage effect, is given by

(9) $$p_{\lambda }^{\prime }\left(\alpha \right)=\lambda \left[I\left(|\alpha |\leq \lambda \right)+\frac{{\left(a\lambda -|\alpha |\right)}_{+}}{(a-1)\lambda }I\left(|\alpha |>\lambda \right)\right],$$

where the symbol I(·) represents the indicator function, and ${\left(x\right)}_{+}=\max\left(0,x\right)$ ensures that only positive values contribute to the penalty.

Unlike LASSO, which applies uniform shrinkage to all coefficients, SCAD selectively penalizes coefficients based on their magnitude. For small values of |α|, SCAD behaves like LASSO by applying an $L_{1}$ penalty to enforce sparsity. However, for larger values, the penalty gradually decreases, thereby reducing shrinkage and mitigating bias. This adaptive behavior allows SCAD to retain important regulatory interactions while still enforcing sparsity, making it particularly suitable for network inference where strong interactions should not be excessively penalized. SCAD was successfully applied for gene regulatory network inference in our recent work [64].

#### 2.3.2. Minimax Concave Penalty

The minimax concave penalty (MCP) is another alternative to achieve sparsity while reducing the bias introduced by LASSO. The MCP penalty function [70] is defined as

(10) $$p_{\lambda }\left(\alpha \right)=\left\{\begin{array}{cc} \lambda |\alpha |-\frac{\alpha^{2}}{2a}, & \mathrm{if}\;|\alpha |\leq a\lambda \;\\ \frac{a\lambda^{2}}{2}, & \mathrm{otherwise}, \end{array}\right.$$

where λ>0 is a regularization parameter that can be estimated using a k-fold cross-validation strategy, and a>1 (typically set to 3) is a tuning parameter that determines the concavity of the penalty. The first derivative of the MCP penalty, which governs the shrinkage effect, is given by

(11) $$p_{\lambda }^{\prime }\left(\alpha \right)=\lambda {\left(1-\frac{\left|\alpha \right|}{a\lambda }\right)}_{+}.$$

Similar to SCAD, MCP applies adaptive shrinkage: small coefficients undergo LASSO-like penalization, while large coefficients receive no additional penalty, reducing estimation bias. This allows MCP to maintain sparsity while preventing excessive shrinkage of large coefficients, leading to more accurate estimates in high-dimensional models. Moreover, MCP is a smooth continuously differentiable concave function, unlike SCAD, which is non-differentiable at certain points. This smoothness often makes MCP more computationally efficient to optimize than SCAD.

Compared to the ridge method, LASSO employs L1-penalization to achieve network sparsity, but this approach can induce abrupt connectivity changes. In contrast, MCP and SCAD utilize non-convex penalties that reduce penalization for larger coefficients, thereby preventing sudden edge additions or removals. By enabling gradual coefficient changes, both MCP and SCAD promote smoother transitions between network stages, resulting in more stable network evolution. This characteristic makes them particularly suitable for modeling gene regulatory networks, where biological interactions typically evolve gradually rather than exhibiting abrupt structural shifts between consecutive time points.

### 2.4. Partial Correlation and Time-Varying Regulatory Network Inference Algorithm

After inferring a directed gene regulatory network using Granger causality, represented by the sparse matrix α, we further classify the regulatory relationships as activation (→) or inhibition (⊣) using the Spearman rank partial correlation method. For each directed edge $X_{j}\to X_{i}$, the activation (→) or inhibition (⊣) relationship is determined based on the partial correlation matrix P, where each element $P_{ji}$ quantifies the direct relationship between genes $X_{j}$ and $X_{i}$ while controlling for the influence of all other genes in the network. A positive partial correlation indicates an activation relationship, whereas a negative partial correlation suggests an inhibitory effect. To extract these relationships, we define a sign matrix $S_{ij}=\mathrm{sign}\left(P_{ij}\right)$, where values of +1, −1, and 0 correspond to activation, inhibition, and no direct relationship, respectively. Finally, we construct a signed adjacency matrix A by performing element-wise multiplication of α and S: $A_{ij}=\alpha_{ij}\cdot S_{ij}$. The signed adjacency matrix A encodes both the sparsity structure (from α) and the direction of influence (from the partial correlation signs), providing a comprehensive representation of the regulatory network.

Algorithm 1 outlines the procedure to reconstruct dynamic gene regulatory networks across different temporal stages using a sliding window strategy. We begin by randomly sampling 80% of single cells and computing the temporal variations of all genes across different time points using various f-divergence measures, as listed in Table 1. This process is repeated 100 times to generate multiple temporal variation vectors. Next, we perform a first-order Granger causality analysis using a VAR(1) model, combined with different regularization techniques to infer sparse directed gene regulatory networks. Finally, compute partial correlations to construct a signed adjacency matrix, where edge signs indicate activation (+) or inhibition (−).
**Algorithm** **1** f-divergence-based dynamic gene regulatory network inference algorithm.**Input:** Time-series scRNA-seq data matrix X; percentage of randomly sampled single cells p=80%; number of samples n=100; f-divergence measures; regularization methods {LASSO,MCP,SCAD}.**Output:** Time-varying gene regulatory networks.**Step 1: Random sampling and temporal variation calculation of genes using f-divergence**Randomly sample a percentage of single cells from the data at different time points.Apply f-divergence to compute the temporal variation, $D_{f}^{j}\left(t_{l}\right)$, for each gene *j*’s expression levels across all single cells between two consecutive time points, $t_{l}$ and $t_{l+1}$, l=1,2,...,n.Normalize the temporal variation, $D_{f}^{j}\left(t_{l}\right)$ with respect to time interval between consecutive time points ($\Delta t_{l}$): ${\hat{D}}_{f}^{j}\left(t_{l}\right)=\frac{D_{f}^{j}\left(t_{l}\right)}{\Delta t_{l}}$.Repeat the above process *n* times to generate multiple temporal variation vectors.**Step 2: Network structure learning using VAR(1) model with regularization**Construct sliding windows that contain $D_{f}^{j}\left(t_{l}\right)$ of two consecutive time points.In each window,
-build a first-order VAR(1) model for each gene *j*:$${\hat{D}}_{f}^{j}\left(t_{l+1}\right)={\hat{D}}_{f}\left(t_{l}\right)\alpha_{j}+\epsilon_{t},$$-Solve the optimization problem using different regularization methods$$\min_{\alpha_{j}}\frac{1}{2}{∥{\hat{D}}_{f}^{j}\left(t_{l+1}\right)-{\hat{D}}_{f}^{j}\left(t_{l}\right)\alpha_{j}∥}^{2}+\lambda p\left(\alpha_{j}\right),$$Output a sparse matrix α that represents the inferred connectivity structure at different stages.**Step 3: Calculate signed adjacency matrix using partial correlation to identify regulatory relationship**In each window, calculate the Spearman rank partial correlation $P_{ij}$ between two genes, and obtain the sign matrix $S_{ij}=\mathrm{sign}\left(P_{ij}\right)$.Construct a signed adjacency matrix A by performing element-wise multiplication of α and S: $A_{ij}=\alpha_{ij}\cdot S_{ij}$.Reconstruct time-varying regulatory networks based on the matrix A.

## 3. Results

In this section, we will apply Algorithm 1 to reconstruct time-varying gene regulatory networks from the time-series single-cell RNA sequencing data, both for in silico and real-world datasets. Specifically, we compare the performance using different f-divergence measures and different regularization terms, including LASSO, MCP, and SCAD.

### 3.1. Datasets and Parameter Configuration

We evaluated the performance of the proposed method using two in silico datasets and one real-world time-series scRNA-seq dataset, consistent with the datasets analyzed in SINCERITIES [40]. The in silico time-series datasets consist of 10-gene and 20-gene subnetworks derived from *Escherichia coli* and *Saccharomyces cerevisiae*, respectively. These datasets have been generated in [40] using a system of stochastic differential equations modeling gene expression dynamics [71], simulated via the Euler–Maruyama method [72]. Each synthetic dataset contains 100 single cells per time point, sampled across eight unevenly spaced time points. For the real-world scRNA-seq data, we used the monocytic THP-1 human myeloid leukemia cell differentiation dataset [73], which consists of eight time points with 120 cells per time point.

In the sparse network inference, the LASSO, MCP, and SCAD regularization methods are implemented using the ncvreg package in R [74]. The optimal penalty parameter λ was selected via 10-fold cross-validation. Self-regularization was excluded from the inferred networks. For the SCAD and MCP penalties, we explored a range of values for the shape parameter *a*, which controls the concavity of the penalty function. To assess robustness, we conducted the full experiment five times and computed the mean values of evaluation metrics, including the AUROC and similarity score, which are described below.

### 3.2. Evaluation Metrics

To evaluate the performance of Algorithm 1, we compared the inferred network edges against a predefined gold-standard gene regulatory network (GRN). We used the area under the receiver operating characteristic curve (AUROC) as the evaluation metric. The AUROC quantifies the model’s ability to correctly identify true regulatory interactions, with higher values indicating better prediction performance. A regulatory interaction is considered a true positive only if both the existence and the sign (activation or inhibition) of the predicted edge match those in the predefined gold-standard network. We compared our results with several state-of-the-art methods, including TSNI [10], GENIE3 [5], and JUMP3 [23], which were also used for benchmarking in [40].

Additionally, we computed similarity scores between inferred networks across consecutive time windows to assess the temporal consistency of the results. The similarity metric is based on edge overlap, defined as the ratio of edges that match, in both presence and directionality (activation or inhibition), the total number of unique edges across the two networks. This metric provides insight into the stability of inferred regulatory relationships over time.

### 3.3. Regulatory Network Inference from In Silico Dataset

**Dataset 1: 10-Gene Data of E. coli.** This dataset consists of a ten-gene subnetwork extracted from the gene regulatory network of *Escherichia coli*, simulated in one-hundred single cells across eight unevenly spaced time points. The dataset is adopted from [40], with self-regulatory interactions excluded from the network. To assess the reliability and stability of the inferred networks using Algorithm 1, the full pipeline is repeated five times for each divergence–penalty combination. The AUROC is averaged across runs, with standard deviations indicating variability.

**Figure 1:**
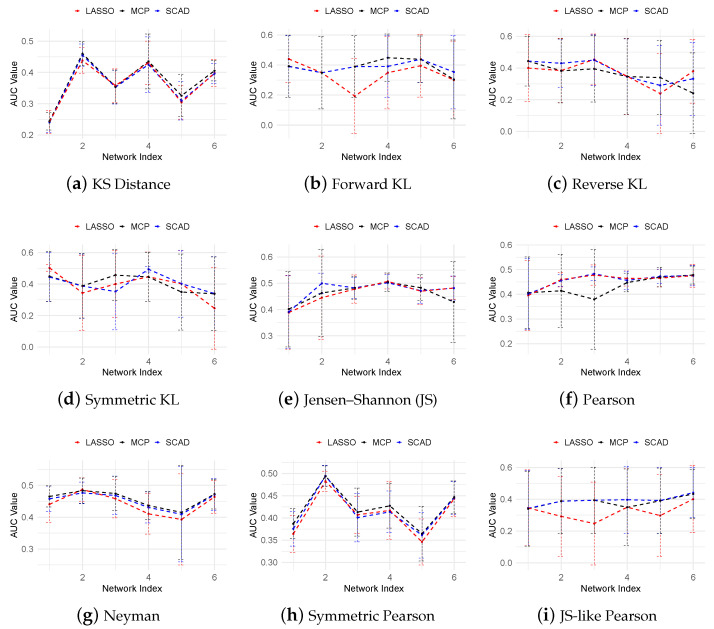
AUROC curves for the 10-gene dataset showing gene regulatory network inference performance across different f-divergence measures using LASSO, SCAD (a=3.7), and MCP (a=3).

Figure 1 presents the AUROC scores for different methods across various f-divergence measures and regularization techniques. The results show that the performance of our algorithm is sensitive to the choice in f-divergence, with Jensen–Shannon (JS) divergence consistently outperforming the other methods. In contrast to previous methods such as TSNI [10], GENIE3 [5], and JUMP3 [23], which infer a single static network from the entire dataset, our approach reconstructs time-specific networks at different stages. According to [40], the AUROC values of TSNI, GENIE3, and JUMP3 range from 0.3 to 0.4, substantially lower than the scores achieved by our method using JS divergence and MCP regularization.

**Figure 2:**
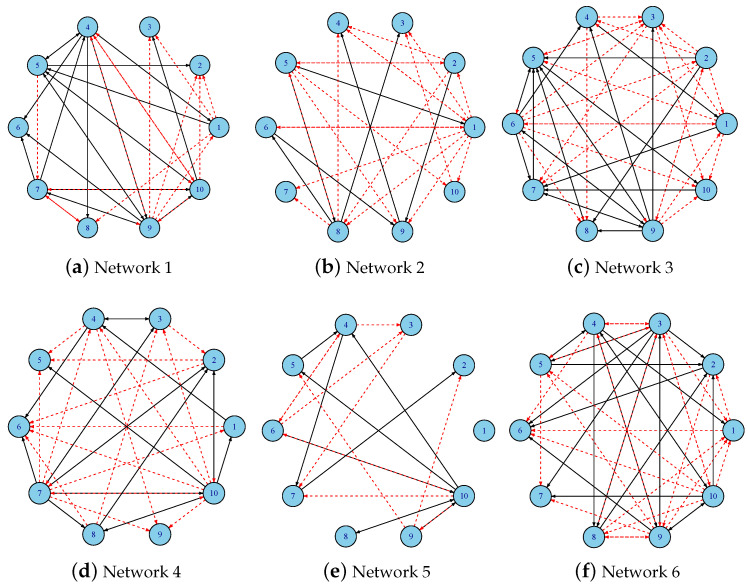
Jensen–Shannon (JS) networks of MCP-based f-DyGRN for the 10-gene dataset with a=3. Each node represents a gene, labeled with its corresponding gene number (1–10). The solid black arrows indicate activation, and red dashed arrows indicate inhibition.

To visualize the inferred dynamic gene regulatory networks, Figure 2 presents six representative time-varying networks generated using MCP regularization with Jensen–Shannon divergence. In each subfigure, directed edges represent regulatory interactions: solid black arrows indicate activation, and red dashed arrows indicate inhibition. These visualizations illustrate the temporal evolution of the network topology and highlight the variations captured by the regularization approach.

**Figure 3:**
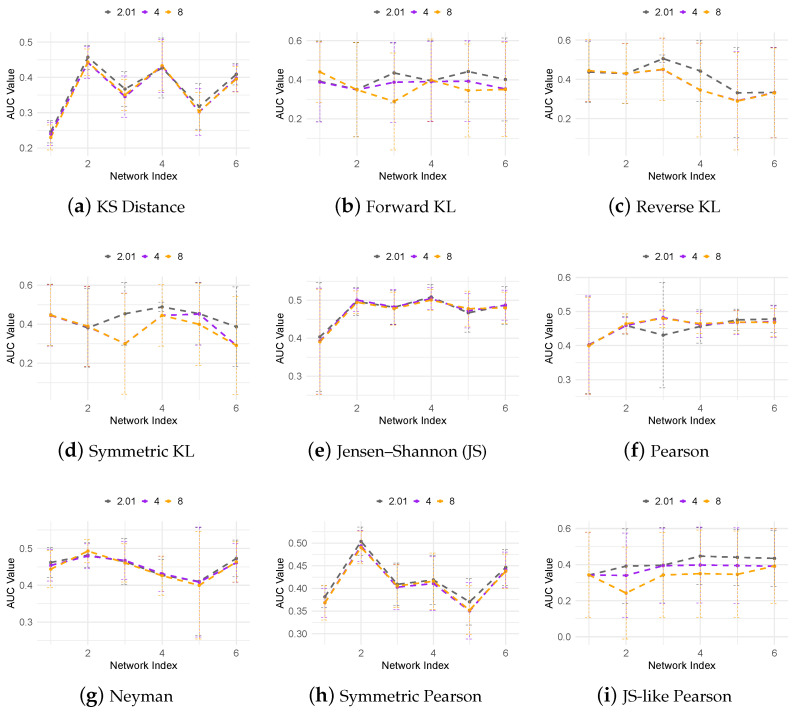
AUROC curves of SCAD-based f-DyGRN for the 10-gene dataset across different *a* values.

Both the SCAD and MCP regularization methods include a shape parameter *a* that controls the concavity of the penalty function. To evaluate the sensitivity of our f-DyGRN method to this parameter, we tested a range of *a* values. Figure 3 presents the AUROC values of SCAD-based f-DyGRN for the 10-gene dataset with a=2.01, 4, and 8 (noting that ncvreg does not permit a≤2; the default is 3.7). The results indicate that the performance is relatively stable across the different KL-divergence variants, while the Pearson divergence family exhibits greater sensitivity to the choice in *a*. A similar evaluation with MCP-based f-DyGRN using a=1.01, 3, and 5 yielded consistent findings.

**Dataset 2: 20-Gene Data of Yeast.** This dataset represents a twenty-gene subnetwork from Saccharomyces cerevisiae (yeast), consisting of one-hundred single cells measured at eight time points. Following the same procedure as in the ten-gene analysis, we evaluated the performance of various f-divergence measures and regularization penalties by computing AUROC scores. With this larger network, our goal was to systematically assess how different divergence–penalty combinations influence the accuracy, sparsity, and stability of the inferred gene regulatory networks.

**Figure 4:**
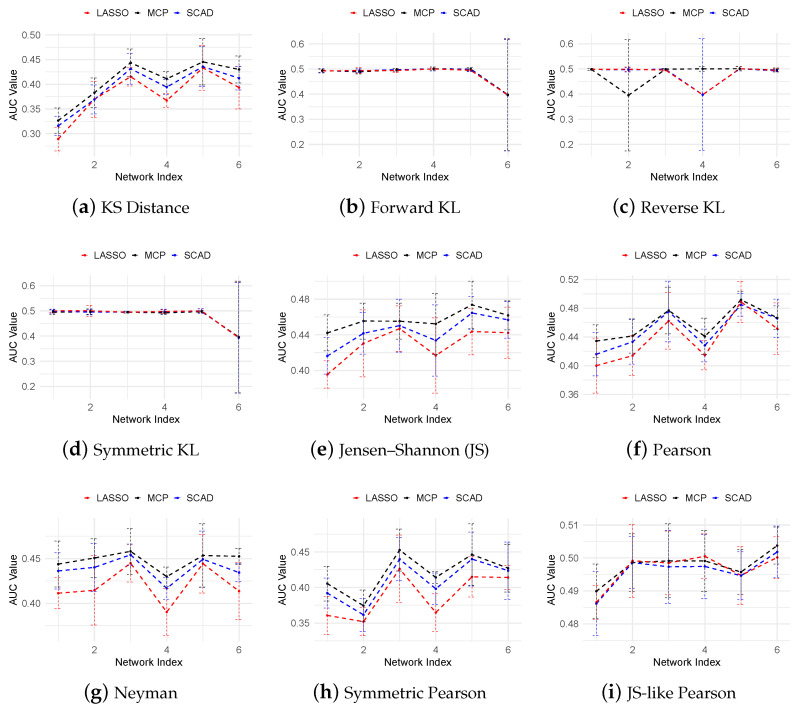
AUROC curves for the 20-gene dataset across different f-divergences using LASSO, SCAD (a=3.7), and MCP (a=3) regularization.

Figure 4 presents the AUROC scores for the 20-gene dataset across various f-divergence measures and regularization techniques, including LASSO, MCP, and SCAD. Consistent with the 10-gene results, the forward KL, reverse KL, symmetric KL, and JS-like Pearson divergences outperform the KS distance used in SINCERITIES, as well as other methods such as TSNI, GENIE3, and JUMP3, whose AUROC values range from 0.1 to 0.4, as reported in [40]. In contrast, most of the Pearson-related divergences perform comparably to KS but exhibit high variability. MCP outperforms both LASSO and SCAD across most divergences, while LASSO consistently shows the weakest performance. Overall, our results highlight the superior effectiveness of the MCP- and KL-based divergences in dynamic network inference.

**Figure 5:**
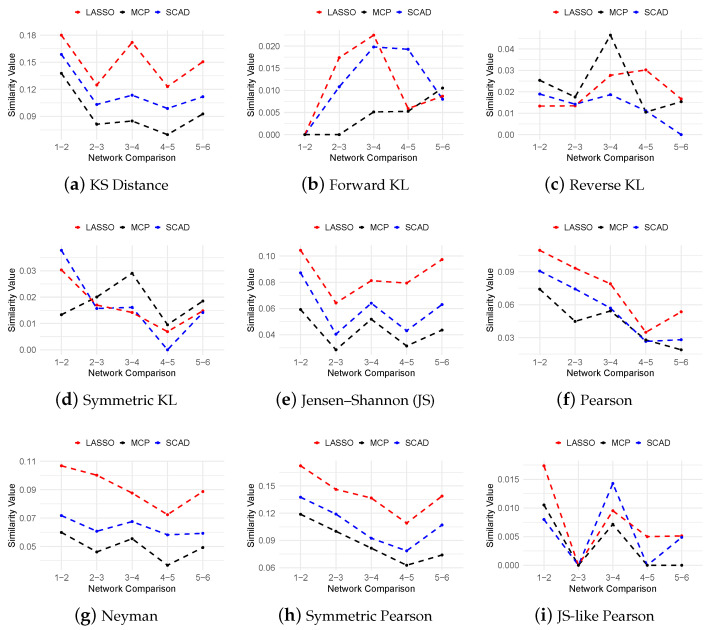
Curves of mean similarity values for the 20-gene dataset across different f-divergence measures using LASSO, SCAD (a=3.7), and MCP (a=3) regularization penalties. The curves illustrate the temporal consistency of inferred networks across consecutive time windows.

Figure 5 presents the mean similarity scores between inferred networks across consecutive time windows, evaluating the temporal consistency of the inferred regulatory relationships across the different methods, f-divergence measures, and regularization techniques. Our analysis reveals distinct performance patterns among divergence and regularization combinations. For the Pearson-based divergences, LASSO consistently achieves the highest similarity scores, outperforming both SCAD and MCP. In contrast, the performance within the KL divergence family varies across distance metrics and penalty functions. The Kolmogorov–Smirnov (KS) distance yields uniformly higher similarity scores across all the regularization methods, with LASSO showing particularly strong performance. These findings highlight that temporal network stability is jointly determined by both the divergence measure selection and regularization strategy, suggesting that methodological choices should be tailored to specific stability requirements.

Similar to Figure 3, we also evaluate the sensitivity of our f-DyGRN method to the parameter *a* in the regularization terms of MCP and SCAD. Although the detailed results are omitted here for brevity, our findings are consistent with those from the 10-gene dataset: the performance of our method is largely insensitive to the choice in *a*. The differences in the AUROC values are negligible, indicating that f-DyGRN is robust to variations in this regularization parameter.

### 3.4. Inferring Dynamic GRNs Driving THP-1 Differentiation

The THP-1 dataset comprises 960 monocytic THP-1 human myeloid leukemia cells measured at eight distinct time points (0, 1, 6, 12, 24, 48, 72, and 96 h) [73] after stimulation by 12-myristate 13-acetate (PMA), profiling 45 transcription factors (TFs) involved in cellular differentiation. Our goal is to infer the dynamic gene regulatory networks that govern the differentiation of THP-1 cells into macrophages.

Following the same experimental setup as used for the in silico datasets, we repeated the full analysis five times for each combination of divergence measure and regularization penalty. For each run, we inferred the regulatory network, then computed the AUROC and similarity scores across the inferred dynamic networks by comparing the results to a gold-standard network containing 20 transcription factors (TFs) identified in [75], which partially overlap with the 45 TFs measured in the dataset. So, our dynamic GRN is inferred using all 45 TFs, but the AUROC calculations are restricted to regulatory edges among the 20 shared TFs. Additionally, we compared our method’s performance with TSNI, GENIE3, and JUMP3, as reported in [40].

**Figure 6:**
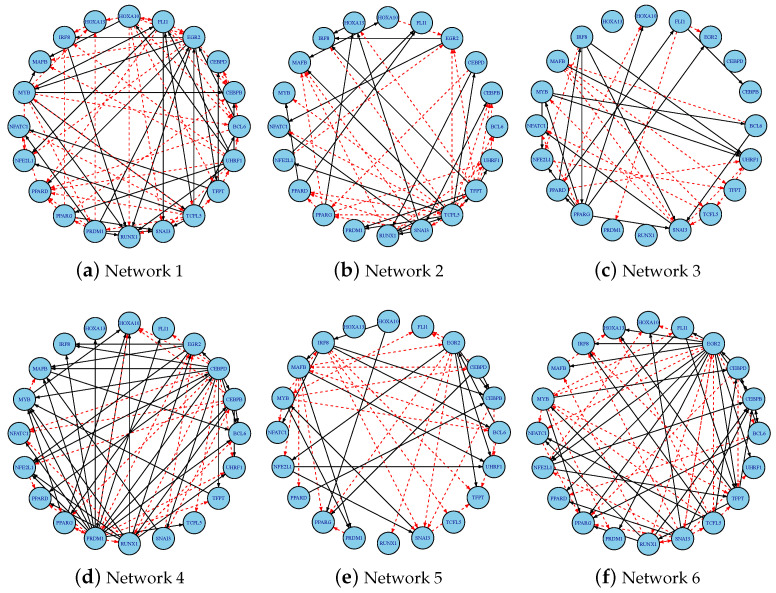
Time-varying gene regulatory networks inferred by MCP-based f-DyGRN using symmetric KL divergence for the THP-1 dataset (a=3). The solid black arrows indicate activation, and red dashed arrows indicate inhibition.

Figure 6 illustrates the time-varying gene regulatory networks inferred by MCP-based f-DyGRN using symmetric KL divergence, capturing the dynamic regulatory mechanisms underlying the differentiation of THP-1 cells into macrophages. Previous methods, including SINCERITIES, TSNI, GENIE3, and JUMP3, were limited to inferring a single static regulatory network using all the available observations and thus could not capture or investigate the dynamic changes in the network structure during the process of cell differentiation. However, our study demonstrates that the inferred network structure is influenced by the choice in f-divergence and regularization method. For example, Figure 6 revealed a stage-specific regulatory relationship between MYB and BCL6: MYB initially suppresses the BCL6 expression during the early differentiation phase, consistent with the reported repressive results in [75], and transitions to MYB-mediated activation of BCL6 in the later stages after PMA stimulation. This temporal switch suggests MYB’s context-dependent dual role in THP-1 monocyte-to-macrophage differentiation, providing a mechanistic basis for PMA-induced phenotypic changes. To facilitate a comparison, we further computed the AUROC scores for the THP-1 dataset across various combinations of f-divergence measures and regularization techniques.

**Figure 7:**
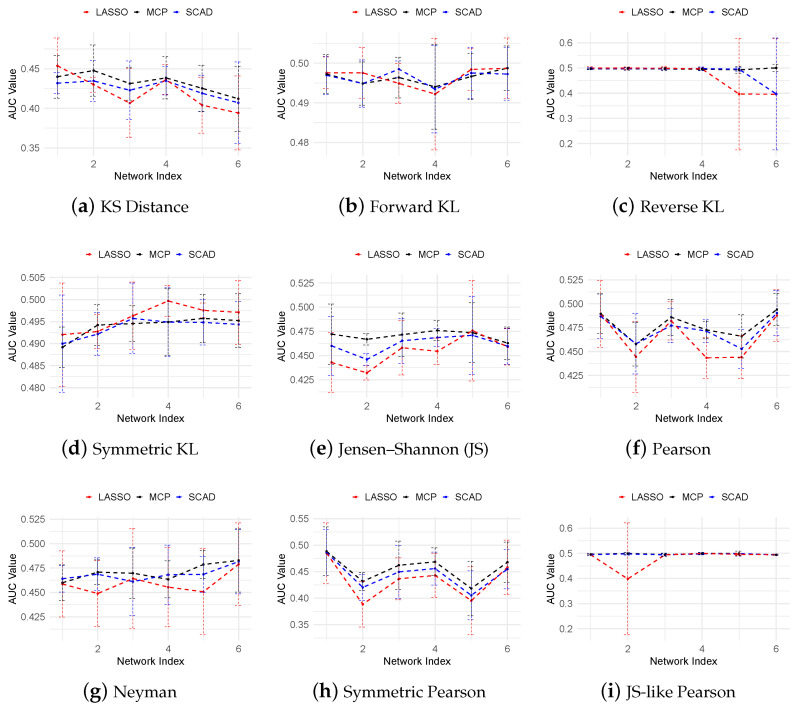
AUROC curves for the THP-1 dataset across various f-divergence measures using LASSO, SCAD (a=3.7), and MCP (a=3) regularization penalties.

Figure 7 presents the AUROC scores for the THP-1 dataset across various f-divergence measures and regularization techniques, including LASSO, SCAD, and MCP. Consistent with the in silico results, MCP consistently outperforms both SCAD and LASSO, achieving the highest AUROC scores, while LASSO consistently yields the lowest performance. Within the KL-divergence family, symmetric KL divergence achieves the best performance. The Pearson divergence family yields AUROC scores around 0.5, slightly outperforming most of the KL-based measures. Overall, our method demonstrates comparable or better performance than TSNI, GENIE3, and JUMP3, whose AUROC scores range from 0.44 to 0.52, as reported in [40].

## 4. Discussion

In this work, we propose a novel f-divergence-based dynamic gene regulatory network inference method (f-DyGRN) to reconstruct time-varying networks from time-series scRNA-seq data. Our approach first employs f-divergence to quantify temporal variation in gene expression between adjacent time points across individual single cells. Next, a first-order VAR(1) model with various regularization techniques is applied to learn a sparse network structure. Finally, partial correlation analysis is used to determine the directionality of regulatory interactions: activation and inhibition relationships. Compared with other state-of-the-art methods, our f-DyGRN approach can reconstruct time-varying gene regulatory networks at different stages, enabling the investigation of how network structures evolve during processes such as cellular differentiation or the cell cycle. While most traditional network inference algorithms require many time points to reconstruct a meaningful network, our method operates effectively with as few as three time points, making it more efficient and applicable to datasets with limited time point availability. We applied this method to both in silico and real scRNA-seq datasets. Our results indicate that the performance of f-DyGRN is influenced by the choice in f-divergence and regularization techniques. The flexibility to incorporate different f-divergence measures and regularization methods allows for a more nuanced investigation of dynamic gene regulatory networks. Our studies found that the symmetric divergences and the Jensen–Shannon divergence consistently demonstrated strong performance, achieving AUROC scores comparable to or exceeding those of benchmark methods such as TSNI, GENIE3, and JUMP3.

Compared to deep-learning-based network inference methods [44,45,46], which suffer from limited interpretability due to their complex neural architectures with numerous parameters, our f-DyGRN framework offers greater transparency while maintaining competitive performance. Moreover, these deep learning approaches exhibit strong dependence on the number of observed time points for accurate inference, whereas f-DyGRN demonstrates robust performance even with sparse temporal sampling. This combination of interpretability and temporal adaptability underscores f-DyGRN’s effectiveness for dynamic network inference tasks.

While f-DyGRN has demonstrated superior performance in inferring dynamic networks from time-series scRNA-seq data, it currently lacks two critical capabilities: (1) detecting temporal change-points in cellular states, and (2) imputing missing values in sparse single-cell data. These limitations present a sequential analytical challenge: given time-series scRNA-seq data with missing values, researchers must first impute the missing entries before applying change-point detection algorithms to identify transitions in network structures. The integration of these capabilities is biologically essential. Accurate change-point detection captures critical shifts in cellular states, while robust imputation ensures the reliability of inferred networks. To address this gap, we propose to integrate the time-series imputation algorithms (tf-BiGAIN [66] or ImputeGAN [76]), advanced change-point detection methods (PLsBD [77] or Finder [78]), and our f-DyGRN network inference approach into a unified framework. This integrated approach will enable simultaneous imputation of missing values, identification of temporal change-points, and reconstruction of time-varying gene regulatory networks at different stages of cellular processes.

## Figures and Tables

**Table 1:** List of f-divergence functions used to estimate the temporal variation in scRNA-seq data. *P* and *Q* represent the distributions of the data with probability density functions p(x) and q(x), respectively.

Name	Divergence Function
Forward KL	$D_{\mathrm{KL}}\left(P||Q\right)=\int_{\Omega }p\left(x\right)\log\frac{p(x)}{q(x)}\;dx$
Reverse KL	$D_{\mathrm{rKL}}\left(P||Q\right)=D_{\mathrm{KL}}\left(Q||P\right)=\int_{\Omega }q\left(x\right)\log\frac{q(x)}{p(x)}\;dx$
Symmetric KL	$D_{\text{S-KL}}\left(P||Q\right)=\frac{1}{2}\left(D_{\mathrm{KL}}\left(P||Q\right)+D_{\mathrm{KL}}\left(Q||P\right)\right)$
Jensen–Shannon (JS)	$D_{\mathrm{JS}}\left(P||Q\right)=\frac{1}{2}D_{\mathrm{KL}}\left(P||M\right)+\frac{1}{2}D_{\mathrm{KL}}\left(Q||M\right),$ $\mathrm{where}\;M=\frac{P+Q}{2}$
Pearson	$D_{\mathrm{Pearson}}\left(P||Q\right)=\int_{\Omega }q\left(x\right){\left(\frac{p(x)}{q(x)}-1\right)}^{2}dx$
Neyman	$D_{\mathrm{Neyman}}\left(P||Q\right)=\int_{\Omega }p\left(x\right){\left(\frac{p(x)}{q(x)}-1\right)}^{2}dx$
Symmetric Pearson	$D_{\text{S-Pearson}}\left(P,Q\right)=\frac{1}{2}\left[D_{\mathrm{Pearson}}\left(P||Q\right)+D_{\mathrm{Pearson}}\left(Q||P\right)\right]$
JS-like Pearson	$D_{\text{JS-Pearson}}\left(P,Q\right)=\frac{1}{2}D_{\mathrm{Pearson}}\left(P||M\right)+\frac{1}{2}D_{\mathrm{Pearson}}\left(Q||M\right)$

## Data Availability

The original THP-1 differentiation data presented in the study are openly available in BMC Genome Biology at [https://genomebiology.biomedcentral.com/articles/10.1186/gb-2013-14-10-r118] (accessed on 11 January 2025). The R code for the proposed method is available on GitHub: https://github.com/yungewang/f-DyGRN (accessed on 6 May 2025).

## Abbreviations

The following abbreviations are used in this manuscript:

scRNA-seq	single-cell RNA sequencing
GRN	gene regulatory network
AUROC	area under the receiver operating characteristic curve
LASSO	least absolute shrinkage and selection operator
MCP	minimax concave penalty
SCAD	smoothly clipped absolute deviation
